# Do ecosystems have functions?

**DOI:** 10.1002/ece3.10458

**Published:** 2023-09-10

**Authors:** Ulrich Krohs, Martin Zimmer

**Affiliations:** ^1^ Philosophisches Seminar Westfälische Wilhelms‐Universität Münster Münster Germany; ^2^ Leibniz‐Zentrum für Marine Tropenforschung, Bremen & Fachbereich 2 Biologie/Chemie Universität Bremen Bremen Germany

**Keywords:** dynamic equilibrium, ecosystem function, ecosystem‐functioning, ecosystem integrity, ecosystem process, ecosystem service

## Abstract

‘Ecosystem function’ and ‘ecosystem functioning’ became core keywords in the ecological literature on ecosystems, their structure, development and integrity. We investigate functions from the perspective of causal contributions to higher capacities, as selected effects, as contributions to the stability and self‐maintenance of organisms and as type‐fixed effects. Based on an in‐depth discourse in philosophy of science, we conclude that ecosystems do not have functions in any sense that goes beyond a mere description of a causal contribution. We recommend the terms ‘ecosystem function’ and ‘ecosystem functioning’ be avoided in the ecological literature (and beyond).

## 1 INTRODUCTION

### 1.1 The use of ‘ecosystem function’/’ecosystem functioning’ in the literature

In the context of the public discourse about perception and reality of both the biodiversity crisis and the climate crisis, science needs to openly communicate with decision‐ and policymakers and the public which, in turn, requires accurate terminology and use of language. With the rise of the social‐scientific concept of ecosystem services and its acceptance in environmental and ecological research of societal relevance over the last decades, more and more studies focussed on ‘ecosystem function’ or ‘ecosystem functioning’ as descriptors of the status of an ecosystem. The use of these terms in the public discourse abounds and they became central elements of the standard vocabulary of every student of ecology. Interestingly, however, the meaning associated with these terms varies tremendously among their users, particularly in multidisciplinary projects and publications, and changed over time. Processes that are driven by ecosystems and lead to providing goods and services that satisfy human needs, either directly or indirectly, have been called ‘ecosystem functions’, for instance, in the often‐cited work of Rudolf de Groot and co‐workers (De Groot, 1992a, 1992b, 1994; De Groot et al., 2000, 2002). This designation is very much based on the human perspective, as it will depend on what humans expect ecosystems to deliver and, thus, reflects an anthropocentric rather than ecological perspective. More recent perspectives often (implicitly) exclude the aspect of services to humankind and refer to ecosystem functions as biotic and abiotic processes within an ecosystem, both in the scientific literature and by international organizations concerned with biodiversity or ecosystem services (e.g. GEOBON, 2023: https://geobon.org/ebvs/working‐groups/ecosystem‐function/; Hölting et al., 2019, 2020; IPBES, 2023: https://www.ipbes.net/glossary/ecosystem‐function; Leuzinger & Rewald, 2021), apparently irrespective of whether or not they lead into anything of use to humans. Thus, by separating natural processes from a human perspective, the ecosystem service concept potentially translates ecological complexity into a limited number of ecosystem services of interest for human well‐being (Costanza et al., 2017; Hölting et al., 2020; Turkelboom et al., 2018). Providing a service is clearly something that is related to human needs: The service does not come to the ecosystem per se, but only in the perspective of its use by humans. If, on the contrary, ecologists speak of an ecosystem function, this can be read as if the function belongs to the ecosystem objectively. Only in an anthropomorphic perspective could this be equated with a service to humans. However, such a perspective has no place in a scientific description of an ecosystem. Hence, it remains unclear what is meant by ‘ecosystem function’ at all.

Even though, thus, the adoption of the ecosystem service concept in ecology could have paved the road towards omitting an anthropocentric perspective from studying and understanding ecosystem processes, confusing ecosystem functions and services is still common in the scientific literature. Despite defining ecosystem functions as ‘properties and processes’ in the recent ecological literature (e.g. Hölting et al., 2019 in their glossary), a direct reference to ecosystem services and their use by humankind still dominates the scientific debate of ecosystem functions (e.g. Wallis et al., 2021, in their critique of a compound geodiversity index as predictor for species diversity; or Zhang et al., 2022, in their editorial to a Special Issue on biodiversity, ecosystem functions and services). We continue to lack a strict definition of the concept of function in this context and its vigorous application in ecological research. Quite the contrary, at least four concepts of function can be found regularly in the ecological literature (Jax, 2005).

According to the above, ‘ecosystem functioning’ sensu De Groot et al. (2002) would be the capacity of ecosystems to do something that is potentially useful to people and mostly refers to the maintenance of energy fluxes and nutrient cycling—ecosystem processes that, in turn, warrant ‘the functioning’ of an ecosystem as entity. This functioning is required for, but not identical with, providing ecosystem services. ‘Functioning’ refers to processes going on in the system. ‘Services’, as we explained above, looks at the outcome of these processes from the perspective of the exploitation of an ecosystem for human needs. If it should make sense at all to speak in addition about ‘the function’ of an ecosystem, this needed to be something in between: not related to human needs like ‘service’, but more than the interplay of the processes involved. One might think about keeping the ecosystem stable as being the function of an ecosystem. While this might sound intuitively appealing, it is at least highly problematic, because it introduces goals like stability of persistence into nature. Concepts referring to goals, so‐called teleological concepts, are absent from the physical sciences since the renaissance. Whether and how their use can be justified in organismic biology gave rise to a vigorous debate in philosophy of biology. Using them in ecology is not less problematic. However, the positions from the debate can be used to spread out the options for concepts of function in ecological theorizing. Based on the in‐depth discussion about the concept of function in philosophy of science, we argue that ecosystems do not have functions, and that the use of the etiologically or even teleologically connotated term ‘function’ for ecosystems and their processes is—at least—misleading. It should be avoided in the ecological literature and, at least as important, also in stakeholder communication. Even the quite common use of ‘function’ in a purely descriptive meaning, counting any process going on in an ecosystem, that is any change in at least one of its variables, as a function, is prone to misunderstanding. The term ‘process’, that is any physical, chemical or biological action or activity that links organisms and their environment, for example, primary production, herbivory, organic matter‐turnover/decomposition or nutrient cycling, would do the job in such cases, without any risk of teleological connotation.

## 2 FUNCTION ASCRIPTION: THE CONNOTATION OF HAVING A GOAL

When speaking about systems and their organization, functions are usually ascribed to components of a system: Organs of organisms are said to have certain functions within, or even for, the organism. Gear wheels are assigned functions within a gearing mechanism, and this mechanism has a function within, for example, a car. The car is ascribed a function only when considered as a component of an overarching system: In the system made up of the car and its users, it helps to achieve the users' goals of getting from A to B (and probably many other goals). On a more general level, it is said having a function within the overall system of transportation. Whether a function is understood as contributing to the operation of an embedding system or else as satisfying some intention: in both cases, it might happen that the function bearer misses to fulfil its task and to meet the goal it is supposed to serve. It may malfunction, that is function not fully or not at all. Function bearers thus can be evaluated with respect to the goals or ends they are supposed to serve (c.f., e.g. Millikan, 1984; Neander, 1991; Wright, 1973). The concept of function allows differentiating conceptually between correct and incorrect performance of the function in question. It is, thus, a concept of the particular class of concepts that discriminate between correct and incorrect, or, in other cases, between good and bad, right or wrong. All these concepts are called normative concepts.

This normative aspect, while unproblematic in everyday language, poses a severe philosophical problem when occurring in scientific contexts. Scientific theories and explanations are supposed to be purely descriptive rather than normative. It would, for example, not be admissible to judge the sun as serving the goals to provide the earth with irradiation and to force it on its elliptical orbit. Though a change in this situation would be unfortunate for life on earth, neither physicists nor biologists would accept describing this scenario as a situation in which the sun malfunctions. Such an interpretation would presuppose that a plan existed somewhere within, or even outside, the universe about how the solar system should be set up properly. This seems nowadays an implausible assumption. Along a similar line, astronomy and cosmology can explain extremely well, supported by highly conclusive observational data, how galaxies and solar systems come into being, change and collapse. We, therefore, know how the present state of the solar system came about and also that it will change so that the earth will become inhabitable in about 500 billion years (if *Homo sapiens* will not drastically shorten this period). Reference to goals and to malfunctioning would add nothing that could improve the scientific explanation, so it seems wise abstaining from it.

However, as said above, the term ‘function’ plays nevertheless an important role in the biological and ecological literature, as it does in technology and in certain sociological approaches. We will first elaborate on the distinction between functions and services, before we elucidate different proposals of how the concept of a biological function can be understood. From the extended debate, we isolate the three fundamental approaches that reconstruct the normative aspect of functions in what is called a naturalist view.

Before discussing these approaches that we count as (1) to (3), we present a deflationary approach (0) that takes function not as related to any goal or norm. Later on, we use these concepts as a toolbox to scrutinize the use of function ascriptions in ecology. For a more comprehensive review of the philosophical debate, see Krohs (2023).

### 2.1 Services versus functions

Ecosystems, their resources, goods and benefits, are used by humans. This is well described and encompassed by the concept ‘ecosystem service’ (Millennium Ecosystem Assessment, 2005) that is human‐centred by definition. This term and its use is well established in the socio‐ecological and socio‐economic literature and meanwhile became central in ecological studies. Since ‘ecosystem services’ is a societal and social‐scientific concept, rather than a biologically or ecologically defined characteristic of an ecosystem, no confusion derives from its use in ecological literature.

Concepts of function, by contrast, whether defined as normative concepts (1) to (3) or taken non‐normatively as in (0) (below) refer to a system considered in its own right, not from the perspective of how humans make use of it. This means that the application of none of these concepts to ecosystems could replace, or even be the basis of, the ascription of ecosystem services. A definition of ecosystem function that identifies functions with, or even only links to, services (see above) levels out fundamental differences between both concepts and, thus, gives up distinctions that help clarifying the subject in question.

We propose to (re‐)enact and maintain this valuable distinction between services of an ecosystem (for us), processes going on in an ecosystem and functions that might or might not occur in systems of certain kinds. Only on the basis of such a distinction can we even ask whether it is ‘the function’ of an ecosystem, or of any of its components, that drives those processes that underlie the provisioning of the above ecosystem services. According to the above line of argument, we answer this question negatively. It is not the function of any component of an ecosystem to contribute to an ecosystem service. Simply the processes going on in an ecosystem happen to do so—no wonder, because the systems are said to provide a service exactly because of its ongoing processes. We also state that ecosystems, with the exception of borderline cases, are not the kind of systems that should be considered to be organized functionally. Their so‐called functioning cannot be judged as being more or less correct; their components do not perform functions, but merely contribute to the processes within ecosystems. Hence, the use of both terms, ‘ecosystem function’ and ‘ecosystem functioning’ is misleading, and they assign characteristics to an ecosystem that an ecosystem does not have. We pledge these terms be avoided in the ecological literature (and beyond). Ecosystems, of course, may show homeostasis, and it might be tempting to consider contributions to homeostasis being functional. As we will show, this resulted in an inflation of functions even in the physical world, where homeostasis occurs as well. A redefinition of the concept of function in this way would be possible, but then we needed another concept for functions in the narrow sense. So, this move would not solve the problem.

## 3 CONCEPTS OF BIOLOGICAL FUNCTIONS

### 3.1 Functions as causal contributions to higher capacities

This concept of function explicitly denies that function ascriptions do have any normative content (Cummins, 1975). It considers any causal contribution of a component of a system to any capacity of the system as being a function. According to this concept, we could indeed ascribe to the sun the function of forcing the earth onto its elliptical orbit. This concept of function is not normative, so any change in the system simply results in new functions. An old function might no longer be present, but it would not make sense to say that it is missing. Nothing would ever be malfunctional. The concept thus allows for functional analysis of any system, but it does not help discerning functional from nonfunctional systems or subsystems. Accordingly, on one hand, the solar system is considered as functional as the hormonal system, and on the other hand, a diseased heart as functional as a healthy one. Any strengthening of this concept that claims that the capacities to which the functions within a system contribute can be identified objectively and refers to the system as a real entity in fact blends a realist attitude with an unnoticed social constructivist approach. The processes described are real, but the delineation of the system and the ascription of capacities are, at least in part, constructions (Jax, 2010, Chap. 5; for a deviating view, see Odenbaugh, 2019).

### 3.2 Functions as selected effects

As the first of the normative concepts of function, we now discuss a concept of function that refers to the causal history—the etiology—of a function as the source of normativity (Millikan, 1984; Neander, 1991). According to this widely accepted etiological approach, the effect for which a trait was selected in evolution is called its function. Thus, if a trait does not produce, better to say, is unable to produce the effect it was selected for under the relevant conditions, it can be said to malfunction. The selected effects approach matches very well to adaptationist evolutionary biology. It denies the idea that other than adaptive mechanisms, like drift or environmental influences on the phenotype, might give rise to functions. Moreover, it makes function ascriptions in the field of physiology, that is in the discipline that focusses on functionality, purely tentative. To really justify the use of the term ‘function’, one would need to demonstrate that a trait was indeed selected for the role that is called its function.

This approach accepts, as is mentioned over and over again in the philosophical literature, that no new trait, that is no trait that was not or not yet stabilized by selection, can have a function, whatever it might contribute to the survival of its bearer, and that no novel effect of any component counts as its function (or as one of its functions). The effect needs to be evolutionarily stable over several generations in order to build up the etiology that makes it a function.

### 3.3 Functions as contributions to the stability and self‐maintenance of an organism

In this second of the normative approaches, an organism is conceived as a homeostatic system, and any contribution of a component to the integrity of the system is considered a function (Mossio et al., 2009). The organism is said to be functionally closed (which poses a problem, for example, for ascribing functions to reproductive organs).

Taking this approach non‐normatively, any change in the system that does not affect its self‐maintenance results just in the appearance of new functions and in the vanishing of others. It would, however, not be adequate to speak about improved or impaired functions. Even when the whole system is about to vanish, no malfunctions can be ascribed, since any standard for functioning properly vanishes together with homeostasis. In this non‐normative reading, the approach collapses into the causal‐contribution approach (0).

As an alternative, this concept comes in a normative version. This requires justifying why homeostasis in living beings differs from homeostasis in other natural systems. Otherwise, we would be obliged to ascribe the functions to rivers and streams, going into and out of a lake, to keep its water level nearly constant, to snowfall and melting to contribute to the homeostasis of a glacier, or to irradiation and heat dissipation of a planet in a solar system. If this attempt (Moreno & Mossio, 2015; Mossio & Bich, 2017) was to be successful, some state of the system would be needed to be defined as its normal state. While there is hardly a criterion within the approach that would allow for defining a norm, the approach could perhaps rely on approach (1) in this regard.

### 3.4 Function as type‐fixed effect

Biological organisms and their traits are described as being members of a species (of a possibly interbreeding population), or as tokens (i.e. instances) of a type (defined, e.g. by the Bauplan, i.e. a set of distinctive characters of a group of phylogenetically related species; Krohs, 2009, 2011). The Bauplan concept has proved for many decades seminal and central to many aspects of evolutionary and developmental biology, and phylogeny and taxonomy (e.g. Willmore, 2012). But why do the atoms and molecules that constitute an individual organism, or—on a larger spatial and organizational scale—cells, tissues and organs (or other structures), arrange in the way that yields exactly this type of organism? The number of possible types of organisms is so large that only a very small proportion of all those possible types is realized. The components do not form the organism in a process of self‐organization: Just mixing the components (atoms, or molecules and ions) would not yield an organism. The types of biological organisms and of their traits are fixed by something like a plan and by (standard) conditions: depending on the biological paradigm, fixation is considered to be brought about by the genome, by epigenetic factors and by what counts as normal environmental parameters. This fixation is fluid and robust at once. It allows for a certain spectrum of instantiations but, nevertheless, for quite high a similarity of most instances among each other. According to the type‐fixation approach, the function of a trait then is its effect that it would have under the mentioned set of plan‐like factors and conditions. A token that deviates too much from its fixed type so that it is not fully performing the function that nondeviating tokens are performing is classified as malfunctioning. Note that it is an empirical rather than a conceptual question to figure out the normal conditions and thus normal functioning. The type‐fixation approach describes the conditions that a theory needs to meet in order to justify function ascriptions in general. Type fixation by selection, as allowed as the only mechanism by the etiological theory (1), is just one possible mechanism. Type fixation by epigenetic changes or by environmental influences are others.

## 4 CONCEPTS OF TECHNICAL FUNCTIONS

Besides in biology, the concept of function is also applied to technical artefacts. Although many technical artefacts are reproduced and thus are candidates for bearing etiological functions (sensu Millikan, 1984), things are not that easy. We certainly want to ascribe functions also to prototypes and to artefacts that are realized only once. Most often, the intentions of designers are taken to set the norm for what counts as functioning. An item on my desk that was designed as a stapler is malfunctioning if it does not staple properly, even if it still can be used as a paperweight. However, users can obviously change the function, and as long as this is done in a rational way (if they have or develop a ‘use plan’, as Houkes and Vermaas (2009, 2010) put it), they might be said to redesign the artefact and, thus, implement new functions on it. Hence, I can make the former stapler a paperweight by developing this (all‐too‐simple) use plan. If it is, sometimes in the future, crashed into pieces, it will become malfunctional even as a paperweight.

Since technical artefacts are in general designed by fixing their type in a construction plan, technical functions can be understood easily by the type‐fixation approach (3). In cases where the construction does not rely on a plan but is completely based on the sequence of constructing steps, like in certain ways of making baskets (Preston, 2013), this sequence fixes the type and, thus, the function (Krohs, 2011). Malfunctioning can again be understood as not showing the effects that were intended and properly implemented by the designer of the system. Likewise, it is unproblematic to define goals for a technical regulatory system, in contrast to the difficulties discussed under (2) with respect to self‐regulatory biological systems. In the technical case, the goal is set by the designer or by the user of the system (e.g. thermostat‐setting in a heating system).

It should be stressed that any application of a concept of a technical function to the nontechnical realm, that is to systems that are not built or modified by humans to meet their own needs, is to be considered being anthropomorphic. In particular, considering the function of the sun being to provide us with radiation of a certain strength and spectrum or to force the earth on its orbit, or considering the function of the global water cycle to provide agriculture with water, would be anthropomorphic. Anthropomorphic uses of the concept of function may help exploiting analogies from technical systems for research into biological and ecological systems, and they certainly help popularizing scientific results. However, they do so for the price of being highly misleading in alluding to intentions where no intentions are present.

## 5 FUNCTIONS OF AND IN ECOSYSTEMS

In this section, we use the tools provided by the philosophical debate about functions to analyse the different uses that are made of function ascriptions in ecology.

### 5.1 External functionality: What is an ecosystem function?

As we have seen, all conceptions of function agree in ascribing functions only to components of systems and never to a system as a whole. This holds even for technical artefacts. They do have their function only as components of an embedding system in which they are, or can be, used. Analogously to this case, assigning a function to an ecosystem, for example the function to protect the coastline from erosion, or to deliver food, wood or other benefits to humankind, conceives it as a component of an embedding system: either of the biosphere, considered itself as being functionally organized, or of human needs and their satisfaction. But in contrast to what talking about the earth or the biosphere as a system might propose, it is not a system that can be said to be functionally organized in any strong sense. The earth system does not strive for persistence, stability, survival or something else. If the system changes, it changes, full stop. Change is undesirable from the human perspective, but not in itself. The great extinctions, though fatal for individuals and populations, were not bad for the biosphere as a system, radiations not good for it. They just happened, the biosphere changed. Judging the earth system, or an ecosystem, from a normative point of view means that the entity which is constituted by species, interactions and processes is inspected through glasses of human perception and expectations. ‘Ecosystem functioning’ then would refer to meeting either our own conceptualization of an overarching system, or even anthropocentric expectations in performance and delivery of functions. We hold that such an anthropocentric perspective does not adequately describe the many processes occurring in ecosystems without providing something to an overarching system and without humans having any relevance therein (or benefit thereof). Descriptions of ecosystems should be neutral in this regard in order to be descriptively adequate. Any anthropomorphic connotation would be inadequate because it adds a dimension that is descriptively unwarranted. Only if one switches from describing an ecosystem scientifically to reasoning about its technical exploitation, goals come into play and neutrality has to be given up. From this perspective, one can talk about the service into which the ecosystem is taken and about the human goal it is subjected to. This exploitation perspective on the ecosystem should—and can easily—be made explicit. Missing to make the change in perspective explicit and hiding it behind talking about functions only leads to confusion.

### 5.2 Internal functionality: In which sense do ecosystems appear functionally organized?

Ecosystems exhibit a certain degree of stability against external stress or disturbance. Such stability includes the resistance of an ecosystem to environmental change, thus, its capability of exhibiting little variability upon environmental change, its rate of recovery from disturbance, that is resilience, and its tolerance to perturbations (Pennekamp et al., 2018). Community composition can be resistant, their processes can be tolerant, and their resilience drives them back to a state of homeostasis that is similar to that observed prior to the disturbance. But nothing goes wrong if this does not happen. Connotating stability of an ecosystem as a positive, and instability as a negative, attribute would follow the anthropocentric perception of change as bad (see above).

Thus, all facets of considering stability as the natural, or even best, state of an ecosystem are closely linked to the concept of ecosystem functioning. However, despite this clear connection of stability to ecosystem function and structure, alike ‘ecosystem function’ itself, the concept of ecosystem stability suffers from divergent views and a multitude of definitions (Van Meerbeek et al., 2021). For instance, ecological systems could reorganize and undergo compositional changes in the face of changing condition while retaining what is considered to be their function and structure (Van Meerbeek et al., 2021; see Lamothe et al., 2019, for an instructive visual presentation of different aspects of ecosystem stability). Moreover, given that many physical systems show homeostasis and stability as well, without this being considered a good reason for describing them in a functional way, these properties of ecosystems should not force us to using the term ‘ecosystem function’ anyway. Homeostasis and stability alone, which occur in many purely physical systems, are not sufficient as a basis for ascribing internal functionality. Even the concept of function as contribution to the stability and self‐maintenance that we discussed above (approach 2) demands more. It explicitly relies on the claim that functionality requires causal closure. Ecosystems, however, are not causally closed.

We would not even evade these problems when weakening the criterion of causal closure and just assign integrity to an ecosystem. The concept of ecosystem integrity has recently been challenged by stressing the contrast between integrity as a measure of wholeness or so‐called naturalness on one hand, and the intrinsic characteristics of ecosystems as being diverse, complex and dynamic on the other hand (Rohwer & Marris, 2021; see Section 6 herein for a brief discussion).

Along this line, it might therefore be tempting to draw the analogy of an ecosystem with an organism and conceive the ecosystem as a functionally organized super‐ or meta‐organism. Some aspects of internal organization seem to point at such a conception: Individual components of ecosystems, that is organisms that belong to species, and their interactions contribute to ecosystem processes. Often, the component that delivers a particular causal contribution can be replaced by another one that takes over this role (multiple realization of functions, c.f., Carrier, 2000). Succession of ecosystems upon disturbance of climax stages can take different trajectories and result in alternative stable or transient states (e.g. Fukami & Nakajima, 2011). In such alternative states, or in seemingly equivalent ecosystem types of biogeographical realms (e.g. temperate steppes—prairie in Northern America, pampa in Southern America, or veld in Southern Africa—versus the tropical savannah of Africa), different species of ecological equivalence can fill these causal roles and drive similar ecosystem processes (Hugget, 1998). On an organismic level, this concept finds its analogue in organs that drive analogous processes in organisms of different species (e.g. protonephridia of acoelomate animals vs. kidneys of vertebrates, both acting in excretion of metabolic waste products). Organs, of course, are more specific to the organism than components of an ecosystem are to the system, so that they cannot be replaced by their functional analogues from other organisms. Nevertheless, they perform the same function in their respective system. By contrast, kidneys do not perform the same function as hearts, and carnivores not the same apparent function as herbivores as drivers of ecosystem processes.

Related to multiple realizations of functions is another characteristic, namely that functions can vary with the context. This is well known from cell biochemistry. The phosphorylation of an enzyme, for instance, can either promote or inhibit its activity; enzymatic reactions are principally reversible, that is, the same enzyme can catalyse both directions of a biochemical reaction, and the predominant direction depends on the context (metabolite concentrations) rather than on the enzyme. This characteristic is known as the heterogeneity of function (c.f., Carrier, 2000). Often, this view is transferred to the ecological realm. Under different environmental conditions, or in different ecological contexts, the same species can drive different ecosystem processes (e.g. omnivorous animals that are either predators or herbivores, or mixotrophs that either perform photosynthesis or consume other organisms, depending on environmental conditions and prey availability). Thus, the role assigned to a species is context‐dependent. Similarly, a single response trait of this species can exert different effects on the environment or on individuals of interacting species. The important difference is that in the case of enzymes we have reason to judge their contribution to biochemical processes normatively and speak of functional vs. dysfunctional molecules. In ecosystems, any reference for such a norm is missing.

Despite the lack of such norms for proper functioning, the perception of species with different roles, or of the succession of species in an ecosystem as a replacement of species that are equivalent with respect to their contribution to the system, has significantly facilitated the spread and development of the trait concept in ecology (e.g. Naeem & Wright, 2003; Tilman et al., 1997; Violle et al., 2007). Within one ecosystem, the co‐occurrence of ecologically equivalent species leads, in a functional view of ecosystems, to functional redundancy that is considered an important fundament of the above‐mentioned tolerance against disturbance.

However, all this is described fully adequately by the causal role function (0), which is *not* a concept that explains anything above the organization of the system. It is misleading to call the causal role a *function* in a context in which functioning is connotated with the idea of matching requirements better or worse, or of the need of fulfilling certain functions. It does not allow for any normative use of the concept: it does neither allow for speaking about mal‐ or dysfunction, nor even to talk about functioning better or worse. Any change in ‘the functioning’ of an ecosystem is merely a change. Even if an ecosystem loses its integrity, it will not become dysfunctional. It merely becomes another—perhaps a less stable—system.

### 5.3 Why do ecosystems not have functions?

So far, we have shown that functions in an ecosystem can only be conceived as causal roles of the function bearers and, insofar, come without any normativity. We now double‐check this result by scrutinizing into the applicability of normative concepts of function to the components of ecosystems. The discussion above provides three possible bases for the normativity of functions: functions as selected effects (1), functions as contributions to stability and self‐maintenance of a functionally closed organism, (2) and functions as type‐fixed effects (3).

None of the above possible anchors holds for ecosystems.

1. The etiological concept, which refers to selected effects, is inapplicable to ecosystems, because ecosystems do not reproduce and thus do not come in lineages of generation. While similar ecosystems may be grouped into types, the similarities between systems of the same type are not due to inheritance. Each system forms and develops spontaneously rather than being the offspring of a predecessor that inherits (some of) its properties. Neither ecosystems nor their components *as* components of these systems are evolutionarily selected. *A fortiori*, these components cannot have selected functions. Our conclusion matches with what is sometimes claimed being the implicit consensus view in philosophy of ecology (Dussault, 2022; Millstein, 2020; Nunes‐Neto et al., 2014). Even co‐evolution, which was put forward as challenging the consensus view (Millstein, 2020), does not lead to functions as selected effects within ecosystems. Their evolution is not based on the selection of lineages of ecosystems (cf., Dussault, 2022), but on the selection of organisms or, in obligatory symbioses, of symbioses of organisms. So, co‐evolved traits are simply selected on the wrong level to make them bearers of functions in an ecosystem. Even in obligatory symbioses, they do only have functions with respect to each other, quite similar to mutually related functions of organs within a single organism, but not with respect to an overarching ecosystem.
2. Whereas ecosystems do show some stability and homeostasis, they do not satisfy the criteria of being (super‐)organisms as required by approach (2). Organs and organizational structures of organisms can be assigned a function, and this perception can be translated into the concept of functional traits of species (e.g. Violle et al., 2007). However, following the above rationale, such a concept is not applicable to the level of an ecosystem, made up by numerous species, in varying combinations, that interact with each other and evolve (as individual species, or in co‐evolution) in response to these interactions and to environmental conditions. This picture does not change if one proceeds to a higher level of abstraction and considers resources, producers, and consumers—perhaps on different trophic levels—being the components of the system rather than particular species. While homeostasis of the system can be observed on this level, homeostatic equilibria can change to new ones, differing from the equilibrium realized in a former state. In its non‐normative version, the approach would have to assign functions to any contribution to any, even to short‐lived, homeostatic state. Functions would change with the system, and talking about functions would collapse into talking about the processes going on in the system at any point in time. As discussed in general above, using such a non‐normative concept of function would be at best superfluous. Taking the concept of function within framework (2) normatively would require spotting a normal state of the ecosystem in question. Given the variability of ecological processes, this will not generally be adequate, and the very concept of ecosystem integrity is dubious (Rohwer & Marris, 2021). Addressing species by their supposed functions, like ‘consumers’ and ‘predators’ mirrors a state of the systems that *is taken as* the normal state, rather than a state *being* normal in the sense of causal closure. The system will not vanish to exist, if a function bearer fails to fulfil its function, but rather reorganize itself. As with any general assessment in the life sciences, there might be exceptions. Some ecosystems might be so strictly integrated that they could be described as symbioses of co‐evolved species quite as well. Such ecosystems, then, could be said to be functionally organized, without challenging our general assessment. An example might be a particular bromeliad ecosystem (*Quesnelia arvensis* and its associated organisms), which is described as satisfying the conditions of functional closure (Nunes‐Neto et al., 2014).
3. While organisms are type‐fixed by a (genetic) plan together with environmental conditions (broadly conceived), where the plan is species‐specific and evolved over generations, ecosystems are self‐organized rather than the realizations of a plan. Different ecologically equivalent species can replace each other over time (throughout succession, or upon disturbance) in any given ecosystem, resulting in alternative states of the system. Thus, even if and when ecosystems can be classified as tokens of certain types, they are not type‐*fixed* in the sense of (3).

The border of an ecosystem is not a well‐defined line or surface, but rather a transition zone (ecotone) into the adjacent ecosystem. Neighbouring ecosystems are not isolated from each other as separate entities, but they are connected and exchange elements, matter (fluxes, e.g. spatial subsidy sensu Polis et al., 1997) and organisms (connectivity). This expansion of the concepts of metapopulation and metacommunity to meta‐ecosystems has been put forward by Loreau et al. (2003) and seeks, among others, to understand the stabilization (see above) of ecosystem processes through interactions at the landscape or regional scale. Following the meta‐ecosystem concept and its implications for fluxes and dynamics beyond ecosystems and, therefore, for what is taken to be ecosystem functioning (Guichard & Marleau, 2021), ecosystems are not even superorganisms, like ant colonies, which show fairly strict delineation against other colonies, are often said to be. Downscaled to the minimalistic version of a biological community, holobionts (Margulis & Fester, 1991), that is assemblages of a host and its symbionts, form a discrete ecological unit within which, however, exchange among connected entities is essential for the integrity of the system. The players (species) in such a system benefit from, and depend on, each other. In this regard, the organization of many holobionts is similar to the functional organization of an organism. Moreover, many holobionts are well delineated, and in some cases, the hosts show even vertical inheritance of the symbionts. It was therefore proposed to abstain from classifying either all holobionts as organisms, or else as mere associations, but to consider *holobiontness* being a gradual property which integrates three dimensions: physical proximity between partners, co‐inheritance and functional integration (Catania et al., 2017). Ascribing functions in the normative sense, then, requires high parameter values in all dimensions of *holobiontness*. An assemblage of organisms that exhibits such a high degree of functional, spatial and genetic integration that the ascription of functions could be justified would then resemble a holobiotic community rather than an ecosystem. An analogous view might be adequate for classifying the *Quesnelia arvensis* system mentioned above as being an ecosystem as well as a co‐evolved symbiotic system. These examples show that functions might be ascribable in some borderline cases, which cannot be classified clearly and exclusively as ecosystems, but belong equally well to other kinds of systems. In order to describe and classify these systems appropriately, we need to consider, among others, the functional aspect. However, the conclusion that functionality is to be considered seriously only in borderline cases supports our result that function ascriptions are inadequate with respect to ecosystems in general.

## 6 CONCLUSIONS AND OUTLOOK

While ecosystems do not have functions and do not function or malfunction, it is obvious from the common and abundant use of these terms in the literature that *some* terms, though not those of functionality, are needed to describe ecosystems and their characteristics in their own right. These characteristics may, but need not, underlie the provisioning of ecosystem services and should therefore neither allude to the possible exploitation of ecosystems nor conceptualize them generally as organism‐like.

We suggest the consistent use of the following terminology:

Replace ‘ecosystem function’ by ‘ecosystem process’, encompassing all courses of (inter‐)actions and activities that result in what is happening in an ecosystem and (potentially, but not necessarily) drives the provisioning of ecosystem services. Since this seems to be the most abundant use of ‘ecosystem function’ anyway, this does not change much—except that it helps avoiding normative connotations that otherwise obscure some debates in science and the public discourse. Along our line of argument, ‘ecosystem functionality’ (not discussed herein), also explicitly referring to the provisioning of ecosystem services (e.g. Hölting et al., 2019), would be best described as ‘multiple ecosystem properties and processes’.

Use ‘ecosystem service’–or, to that end, ‘goods and benefits’ or ‘nature's contributions to human wellbeing’, as in the more recent body of literature—for everything provided for society and humankind by the ecosystem processes that occur within an ecosystem.

In the recent literature, ‘ecosystem functioning’ has often been synonymized with ‘ecosystem integrity’, while there is no commonly accepted definition of this term either (Bridgewater et al., 2014). In a recent critique, Rohwer and Marris (2021) stressed ecosystem integrity be ‘neither real nor valuable’, because it merely reflects a human perception of, and wish for, ‘wholeness’ or ‘naturalness’ of an ecosystem, while the very characteristics of ecosystems are diversity, complexity and dynamics in space and time, rendering them ‘unlikely to possess “integrity”’. This critique of an anthropocentric perception of ecosystems certainly goes in line with our argument. While Karr et al. (2022), in their reply to Rohwer and Marris (2021), refer to the concept of ecosystem disintegration, meaning the loss of integrity, as being central to ecosystem conservation and re‐establishment (*sensu* Zimmer et al., 2022) and even to our very understanding of human impacts on ecosystems, we follow Rohwer and Marris (2021). We propose to replace ‘ecosystem functioning’ by ‘dynamic equilibrium of an ecosystem’, as describing the dynamic status of an ecosystem that allows for ecosystem processes to occur and to drive the provisioning of ecosystem services needed by society and humankind worldwide. Even when understanding ‘ecosystem integrity’ as denoting a status of an ecosystem that allows for natural diversity, complexity and dynamics in space and time, as well as for resistance, tolerance and resilience to (anthropogenic) stressors and disturbance, we certainly agree with Rohwer and Marris (2021) in that we should be explicit with what and which aspect of integrity we are talking about.

## AUTHOR CONTRIBUTIONS

**Ulrich Krohs:** Conceptualization (equal); formal analysis (equal); investigation (lead); validation (equal); writing – original draft (equal); writing – review and editing (equal). **Martin Zimmer:** Conceptualization (equal); formal analysis (equal); validation (equal); writing – original draft (equal); writing – review and editing (equal).

## Data Availability

Data sharing not applicable to this article as no datasets were generated or analysed during the current study.
